# The Pathology and Treatment of Hemorrhage from Mucous Surfaces

**Published:** 1858-04

**Authors:** E. Read

**Affiliations:** Terre Haute, Indiana


### *The Pathology and Treatment of Hemorrhage from Mucous Snrfaces.*

By E. Read, M. D., Terre Haute, Indiana.

No tissue in the human organization is so incident to, or is so frequently the source of hemorrhage as the mucous. Hence we have as common maladies, epistaxis, hemoptysis, hematemesis, hematuria, hemorrhoids, menorrhagia, &c. No part of this tissue exempts it from this common hemorrhagic law. Some portions, however, are not only more liable to it in frequency, but in danger.

Hemorrhages are at all times alarming, both to patients and friends, and are, not unfrequently, the most formidable ailments, which fall to the charge of the physician.

I propose in this paper to examine the pathology and treatment of mucous hemorrhages. I have stated above, that these surfaces are exceedingly liable to hemorrhages, and among the most prominent reasons for this frequency, may be cited the liberal supply of blood to this surface, and the great excess of veins over the arteries, the disparity here being much greater than elsewhere. When in a state of health, however, the venous blood courses these mucous channels safely and faithfully, and only fails to do so, when antecedent or co-existing obstructions prevail, inducing a *termination* in hemorrhage from capillary distension. When the circulatory system is equally balanced, when no obstructions exist, when the conveying and conveyed parts are in a healthy state, hemorrhages are not to be anticipated, but when the reverse obtains, blood may be poured out, giving the different varieties of hemorrhage before mentioned.

In all these cases, the blood is venous, and where it is altered from previous disease, or from debilitated habit, its flow is more profuse, and its arrest more difficult, because in such habits the congestion is more intense, which forces a greater capillary distension, and an easier rupture, and a more ready exosmosis.

In such cases the fibrin of the blood is diminished, its coagulability is lessened, and the venous coats have shared the general debility, which facilitates their rupture; hence expistaxis not unfrequently becomes dangerous in habits, with enlarged spleens and loins, and those suffering from general malarious prostration.

I lay down then the general proposition, that these hemorrhages are the result of congestion, and that the basis of the treatment is to be found in anti-congestive remedies, and while I shall maintain this proposition as a whole, I shall speak of other causes, which have and ought to be recognized as inducing hemorrhage from the mucous membranes.

These hemorrhages may result from general plethera—such is epistaxis, especially when it takes place in the young and robust, and apoplexy, when it is found in those of full habit.

They may result from the intropulsive operation of cold. But last year I was called to treat in a single very cold winter's day, two persons, but a little beyond the middle of life, who had apoplectic strokes, one of whom died, never having spoken after he fell.

In this case, blood was evidently poured upon the brain from the rupture of a blood vessel, the result of intropulsion of blood from cold.—From the same cause, epitaxis may also take place.

Hemorrhage of these surfaces, may likewise result from irritants. Hematemesis may follow the introduction of irritants into the stomach, and hematuria arise from stimulating diuretics and hemoptysis from the inhalation of irritants upon the lungs.

Atony of the blood vessels may also cause hemorrhage by congestion, and in this state, it takes place in the dependent parts. The erect posture may bring on fatal uterine hemorrhage in debilitated subjects with this diathesis, and a stooping posture may induce apoplexy.

A general or local hyperemia usually precedes hemorrhage—that is, blood is determined to a part in an active and uninterrupted manner, which may give rise to active hemorrhage, or there may be excess of blood in the vessels of a part, with a diminished motion of that blood, and this forms what is called passive hemorrhage.

I exclude from present consideration all hemorrhages resulting from mechanical injury, which frequently may arise from blows and strains.

It is scarcely reasonable to suppose, that hemorrhage depends altogether upon hyperemia; but that, in most cases, there is in addition an altered or diseased condition of the contained and containing parts, that is, of the vessels themselves, and of the blood circulating in them. I can not doubt, but that in many instances, both are diseased or altered, but in most cases, that the vessels themselves are only weakened from a prëexisting general disease, while the great change exists in the blood itself. It is more fluid—less coagulable—is defective in fibrine, and is just in that condition, that the smallest cut or breach of texture, admits of the most troublesome oozing of blood. This may be termed the hemorrhagic diathesis, and is recognized by a pallid or sallow hue of the skin, and a paleness of parts naturally covered with blood.

In many diseases, the blood is not only deficient in coloring matter, but the coloring matter is changed, as is known to be the case in malignant fevers. Such was observed in the Walcheren fever, where the blood was said to have changed to a pitchy black. In cachexia, from malarious influence, and disease of the spleen, the blood is poor and perverted.

Petechiæ and ecchymosed patches in malignant and low forms of fever, are probably owing to a change in the coloring matter in the blood, and to a breaking up of the red particles. The various shades and unhealthy hues observed among the poor and ill fed, and the cadaverous cachectic sallowness of those residing in malarious regions, may be ascribed to a change in the coloring matter of the blood.

The changed condition of the blood and of the blood vessels are co-existing. Firmness and elasticity and density can not exist in the vessels, when the blood is thin and poor, and wanting in every healthy element to render them so. If it were possible to have them in a perfectly healthy state, the thin and watery blood might be carried along without transudation. A French physiologist observed from experiments which he made, that animals from whose blood fibrin had been abstracted, were easily effected with congestions and effusions upon different organs, and this may be ascribed to what another Erench philosopher regarded a general physical fact: that very thin fluids pass with greater difficulty through capillary tubes, than those of greater spissitude.

It would seem to be reasonable, that a fluid with cohesive properties would be driven with greater facility through the hydraulic apparatus of the circulation, than that which is thinner, and that the latter condition may be more favorable to irregularities in the distribution of the blood. It is thus that we may have the most intense cerebral pain from great loss of blood. It is thus that the pale and anemic female suffers so much from headache, which can alone be relieved by tonics and generous diet. Hence follows the proposition, that the weak and debilitated, the poor and ill-fed and clad, the old and the infirm, and in fact, all whose blood runs white instead of red, are the fittest and most frequent subjects of congestions. Is of them, that the cholera is every where fed, in all the great marts and thoroughfares, wherever it has passed. It is of them, that intermittents are made up, where congestion alternates in every organ in the human system. It is of them that uterine congestions and hemorrhages are made up.

It is of them, the old, the infirm, the debilitated and cachectic, whose blood is deficient in every healthy element, in red particles, in fibrin and albumen, that are drawn the subjects of pneumonia and pulmonic congestion.

It is of them that mucous hemorrhages come, supervening congestions, and forming what is usually denominated passive hemorrhages.

Now most hemorrhages are of this class. Active hemorrhage, as stated heretofore, may and does sometime exist, but rare however, compared with the passive.

When the whole system in all its various parts and functions, is in a state of health,—when the blood vessels are firm—when the blood is rich in all its essentials, when it is as new wine in new bottles, there is but little danger of hemorrhage of an alarming character, except in the rupture of a brain vessel, when a small quantity would cause death. In such habits, congestion rarely takes place, and consequently hemorrhages rarely.

But in the cachetic—in the pale and sallow and debilitated—in those who bear upon their external the impress of decay, how liable are they to congestions! And when the blood is arrested at any point, and there accumulates, and there is not vitality euough to propel it along, the thin blood oozes through the relaxed blood vessels, and we have a hemorrhage difficult to check. The blood vessels of the skin itself may give way, but the more delicate ones of the mucous surface, are those from which the great danger is to be apprehended. How often do we see the delicate female succumb to uterine hemorrhage? How often do most vexatious hemorrhages gush forth from the schneiderian membrane? How often do the gums become spongy, relaxed, and hemorrhagic? How often does the extraction of a tooth endanger the life of those of this tendency, and how often do we encounter purpura hemorrhagica?

I have said these hemorrhages, epistaxis, hematemesis, hemoptysis, hematuria, menorrhagia, and albuminaria, may arise in full plethoric and heal[illegible] ubjects, and may be active, but by far the greatest number are passive, and the subjects of these are feeble, pale, and dissolving.

Wherein the transit of blood is interrupted in its circuit in any organ, the effect of that arrest may be expended in a remote part; for instance, splenic congestion gives rise to epistaxis—hepatic congestion to hemorrhoids and dysentery.

In all malarious districts, these hemorrhages are common for the reason that congestions are so, and they are concomitants of congestion. Do they arise from inflammation?

I have no hesitation in declaring my belief that inflammation of the mucous surfaces does not terminate in hemorrhage. I have no hesitation in the declaration, that none of the above hemorrhages, as a rule, result from inflammation.

Can it be anywhere shown, that antiphlogistic remedies are applicable to these cases? Can it anywhere be shown from good authority, that these surfaces pour out blood from inflammation, either during its existence or termination, and yet we are told that antiphlogistics are the remedies to be used.

In those parts of the mucous tissues exposed to visual inspection, did any medical gentleman ever see inflammation terminate by the pouring out of blood? We cite the mouth, fauces, nose, eyes, and vagina, as examples.

I wish especially to impress the proposition, that such results are never anticipated and never found; but that these hemorrhages are the result of congestion, which congestion mostly takes place in weak and debilitated subjects, with impoverished blood. I hope those who may do me the favor to read these pages, will reflect upon this matter carefully, and if it can be done, will point out the written authority or the personal experience, proving that inflammation of mucous surfaces does terminate in the effusion or transudation of blood.

I particularly invite investigation upon this point, because if the general law of the termination of inflammation of these surfaces does not sanction such effusion or transudation, I shall then have established additional evidence of the non-inflammatory condition of dysentery, the pathology of which I have endeavored to study carefully and without prejudice. And here let me disclaim all partiality or fondness, for any doctrine, however ancient and long received, or however new and fascinating, which will not bear the test of experience and of careful examination. I have been taught from the saddest experience, the necessity of a change of practice in many diseases, and especially those which have hitherto been regarded inflammatory—the treatment of which, under that view, was insufficient and unsatisfactory; but which have been controlable under remedies applicable to congestion.

Of these may be mentioned dysentery, pneumonia, and hemoptysis. In addition to what has already been said upon this subject, I may state that hemorrhages prevail mostly in those seasons when malaria is most rife, and in these countries where we have most agues.

I have particularly observed this of epistaxis and hematemesis. Every year, towards the close of summer and in the fall, I see a great deal of epistaxis, and what has been particuly a matter of observation with me, is, that the very first frost of fall, largely increases it both in numbers and in formidableness. It mostly occurs too at night, and am sure of being deprived of my rest on that, if on no other night.

In 1845, more persons suffered from congestions in this region than before or since, and during that summer, I saw not less than twenty persons, who, upon the invasion of a chill were seszed with hematemesis, and who vomited large and alarming quantities of blood. One and two quarts were usual measures.

During this, the surface was cold, chilly, and death-like, and the thirst intense and unquenchable. In these cases, the invasion was sudden ; the blood was poured out into the stomach from ruptured mucous capillary vessels, the result of a chill of congestion. In no way could it arise from inflammation.

In all such, I carefully withheld cold drinks, and gave in their stead hot gruel in table-spoonful doses every few minutes, in conjunction with hot whisky or brandy toddy, and applied mustard cataplasms over the epigastric region, and hot applications to the extremities and surface generally.

In addition, I gave x grs. quinine immediately, and v grs. every hour afterwards until reaction was restored, and the pulse became full and the surface hot. Undar the free use of tonics and stimulants, the bleeding was readily checked, and its return prevented. I have impressed upon my mind one case which I saw accidently. I had visited the family in the morning, two or three were sick. As I was near by in the evening, it occurred to me that I had better call, and see if all was well with my patients. In addition to those already sick, I found a stout young man had two hours previously been seized with hematemesis, and as I myself and other physicians were all absent, he had not been prescribed for He suffered no pain—said he felt quite well, except weak, but was intensely thirsty—cold drink was all his desire. He was pulseless, very cold, with a cadaverous odor, and seemed in a collapsed and moribund state. I gave him 15 grains quinine immediately in hot brandy, suspended his cold drinks, had hot gruel provided, immersed his hands and feet in water as hot as could be borne, and remained with him until reaction was restored. He recovered.

About the same time, I was called to visit a stout laborer, 32 years of age, who was bleeding profusely from his bowels. An hour previously he had been seized with a chill, and immediately the hemorrhage supervened. He was cold and pulseless, and the blood poured from him when lying in the most quiet manner. I had never before, nor have I ever since, seen a case so alarming and apparently so desperate. Immediately I gave him x grs. quinine in conjunction with hot brandy toddy, and applied external warmth and mustard cataplasms over the bowels. In an hour's time, reaction was restored, the bleeding ceased, and under the free use of tonics and stimulants he recovered. He was intensely thirsty, and was not satisfied with any but the coldest drinks. As reaction took place, the hemorrhage ceased, and there was not a recurrence of either chill or bleeding. In this case and in all others, where the invading chill is severe, or where it is complicated with untoward symptoms, tonics must be administered promptly and boldly.

A timid and undecided treatment will surely result in death. In the case just cited, what other treatment could have offered the smallest hope of relief and restoration to health?

No one opinion in medicine was ever so absurd, or has been productive of so great a loss of life, as that which inculcated the doctrine, that the tongue must be made clean by alteratives and cathartics, before tonics could be safely used. In most cases, where malaria is abundant, and where other combinations render the system peculiarly liable to its impression, death would supervene before the time would arrive to use tonics under that belief. Not only this, tonics will clean the tongue much sooner and better than any or all other remedies combined. From a long experience in intensely malarious regions, I am pursuaded that in most cases tonics may be used at once and freely without any preparation whatever.

By a prompt tonic treatment, the disease may be cut short and organic lesions prevented.

Treatment of Mucous Hemorrhages.—It is obvious from what I have heretofore said, that I regard most mucous hemorrhages to be of a congestive character—hence I discard in their treatmeut, blood-letting, nauseants, and astringents.

Epistaxis is a very usual concomitant of ague, and is sometimes very troublesome. Formerly I was in the habit of using cold applications freely, and have seen the blood pour out without remission and to a dangerous extent, when my patient was already pale and almost pulseless, and was cold and chilly. I have used styptics and have plugged the nares anteriorly and posteriorly, and still the blood ran. It was devoid of coagulability—it had no fibrin. I have used nauseants, and still it was not checked. In the treatment of this kind of hemorrhage, the only rational and safe practice is, to remove the congestion and to equalize the circulation. This is to be accomplished by a liberal use of tonics and stimulants, and the application of external warmth. In no instance have I failed to arrest the hemorrhage promptly, when I have given from five ten grains quinine, and repeated as required, together with stimulants and the application of external warmth. Cold and astringents and styptics, and nauseants are absolutely hurtful. This treatment applies to every mucous hemorrhage, as well to epistaxis as to any other.

Several years ago, a thin, spare bachelor quaker, named John Hare, kept a hotel near the old Court House in Cincinnati. He was very subject to hemoptysis, and having an office convenient, I was frequently called to prescribe for him, when he was bleeding, which happened mostly at night. Faithful to the doctrines which I had been taught, I invariably bled him copiously from his arm, and depleted him otherwise, *pro re rata*, and in the most approved manner. In spite of my treatment, my quaker patient lived, and for aught I know may still live. My practice had no arresting power. Periodically he bled.

I trust I have become wiser in the treatment of hemoptysis, and that I prescribe more in accordance with its true pathology. During the hemorrhage, I apply a large and warm mustard cataplasm to the chest, administer quinine and stimulants freely, and apply warmth to the extremities. During the interval, quinine, bark, and stimulants, either brandy or whisky, and a general tonic course is to be pursued. In other words, the whole system is to be invigorated. With this treatment, I am persuaded I have saved very many, who would under any other, have gone down to the grave surely and speedily. Six years ago, Dr. Young, my partner, a young man, without any premonition except a fullness of the lungs, was seized with a violent and alarming hemoptysis.

Within a few weeks it occurred twice, but under the liberal use of tonics and whisky, he entirely recovered.

For two years, however, he used quinine almost daily, and whenever he felt a pulmonic fullness with an irritating cough, he co[illegible] check it at once with a few grains of quinine. He has entirely recovered, and is robust and healthy. I could record many other similar cases.

In every species of mucous hemorrhage, our main reliance should be upon the liberal use of tonics and stimlants, in connection with a generous diet. The system should be invigorated—irregular distributions of the blood prevented—and local hyperemia discussed.

No one of these hemorrhages requires a more liberal use of these remedies, than does menorrhagia. Its victims are the pale and feeble and bloodless, and the doctrine which teaches the use of other remedies, should at the same time teach a language to console the sorrowing hearts of those, who are called to mourn departed friends.

I have already written, I hope, at sufficient length, to attract the attention of the profession to the subject I have discussed. In all alarming hemorrhages, the physician must be fortified with suitable remedies, and with the proper reason for their use. In such cases, there is usually too much confusion for quiet thought.

I am persuaded that no class of diseases, have generally been so badly treated as these. Depletion has been used when tonics were the only proper remedies, the blood has been made thinner and poorer, when it should have been made thicker and richer.—*Cin. Lancet & Observer.*

